# Pericardial Disease in Cancer Patients

**DOI:** 10.1007/s11936-018-0654-7

**Published:** 2018-06-23

**Authors:** Arjun K. Ghosh, Tom Crake, Charlotte Manisty, Mark Westwood

**Affiliations:** 1 Cardio-Oncology Service, Department of Cardiology, Barts Heart Centre, Barts NHS Health Trust, St Bartholomew’s Hospital, West Smithfield, London, EC1A 7BE UK; 2 Cardio-Oncology Service, Department of Cardiology, University College London Hospital, 235 Euston Road, London, NW1 2BU UK

**Keywords:** Cancer, Pericardium, Cardio-oncology

## Abstract

**Purpose of review:**

To understand the variety of conditions in which the pericardium may be affected in cancer patients.

**Recent findings:**

Cancer may affect the pericardium directly (primary cancer; uncommon) or through metastases (commoner). Cancer treatment (chemotherapy and radiotherapy) may affect the pericardium leading to pericarditis and myopericarditis. Pericardial effusions, tamponade and constrictive pericarditis are complications that can also occur. A variety of techniques (predominantly cardiac imaging related) are used to make the diagnosis with the treatment strategy dependent on whether the pericardial disease is due to cancer or as a result of cancer treatment.

**Summary:**

A variety of pericardial diseases may be caused by cancer and cancer treatment. Determining the aetiology and providing effective treatment can often be challenging.

## Introduction

Pericardial disease in cancer patients commonly manifests in one of two ways. It may be a manifestation of the disease itself, e.g. malignant disease affecting the pericardium either directly or via a pericardial effusion or as a result of treatment (chemotherapy or radiotherapy). The aetiology of pericardial involvement can be difficult to determine (due to the dangers inherent in pericardiocentesis and pericardial biopsy) and the management of certain conditions (recurrent pericardial effusions and constrictive pericarditis) can be very challenging [1]. This review will cover the different types of pericardial disease in cancer patients and the associated management options.

## Pericarditis

The pericardium consists of two layers—the outer fibrous pericardium and the inner serous pericardium. The serous layer is composed of the outer parietal layer fused to the fibrous pericardium and the inner visceral pericardium which is fused to the epicardium. In between the parietal and visceral layers is the pericardial space which contains the lubricating pericardial fluid (around 50 ml normally).

Pericarditis is the commonest form of pericardial disease [2, 3]. It usually affects young and middle-aged people and often recurs [4]. It is responsible for around 5% of presentations to emergency departments with non-ischaemic cardiac pain [5]. Mortality rates can reach 1%. The aetiology is often unclear as many cases are undiagnosed. Interestingly, pericarditis may be a marker of occult cancer with over 10% of 13,759 individuals with pericarditis subsequently going on to be diagnosed with cancer in a Danish registry [6•]. The increased risk of cancer persisted for over a year in those with pericarditis and the overall prognosis was worse for these individuals who went on to be diagnosed with cancer. Cancer-related causes include primary tumours (e.g. pericardial mesothelioma (rare) or secondary tumours (lung and breast primaries and lymphoma)).

Radiotherapy-induced pericarditis occurs acutely in animal models [7]. The underlying cancer lesion and as such direction of radiotherapy beam can influence the incidence of pericarditis with an increase in incidence ratio of 1.61 (1.06–2.43) in left-sided breast tumours compared to right-sided breast tumours [7, 8]. While radiotherapy-induced pericarditis can occur early [9], the maximal incidence was at 5–9 years in a study of patients receiving radiotherapy for breast cancer [10]. The treatment of other cancers with radiotherapy can lead to pericarditis with this being reported a few weeks after therapy in Hodgkin disease and oesophageal cancer [11]. With longer follow-up going forwards, the incidence of radiation-induced pericarditis may change.

The dose of radiation is important. In one review, >50% of patients who received > 30 Gy of radiation for a variety of diseases (Hodgkin and non-Hodgkin lymphoma) developed pericarditis [11]. Other studies have focussed on the area of the heart involved showing that radiation-induced pericarditis is more likely if a large proportion of the heart (> 30%) receives a dose of at least 50 Gy [12].

**Fig. 1:**
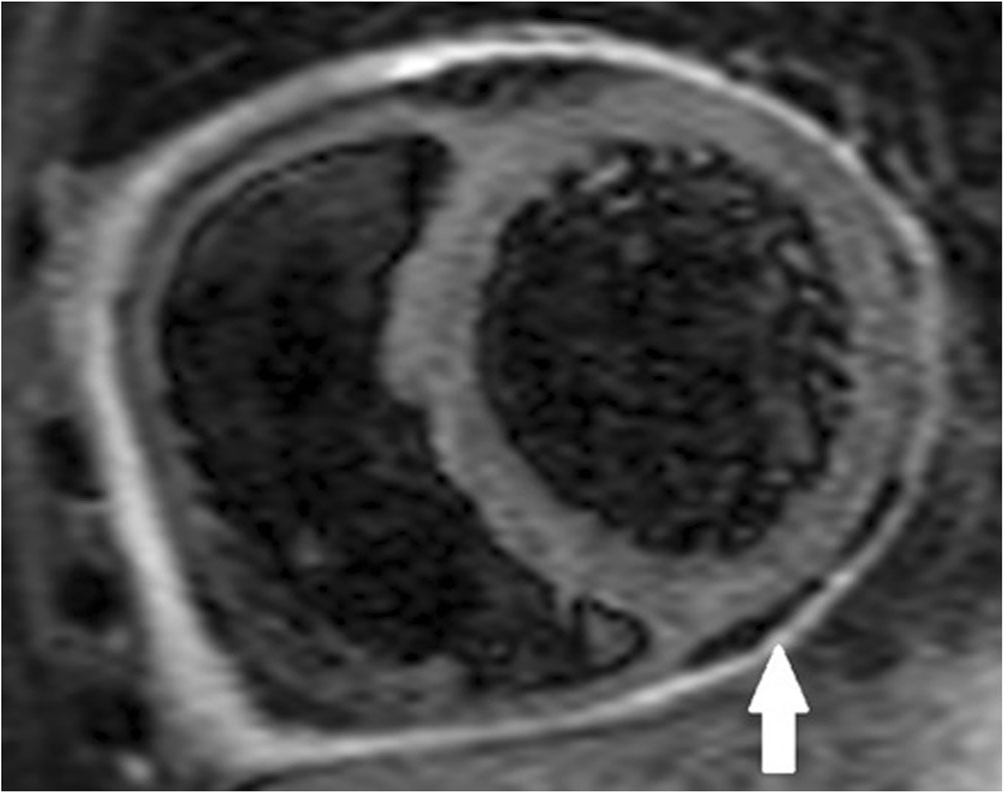
STIR CMR image demonstrating pericardial inflammation (white arrow).

**Fig. 2:**
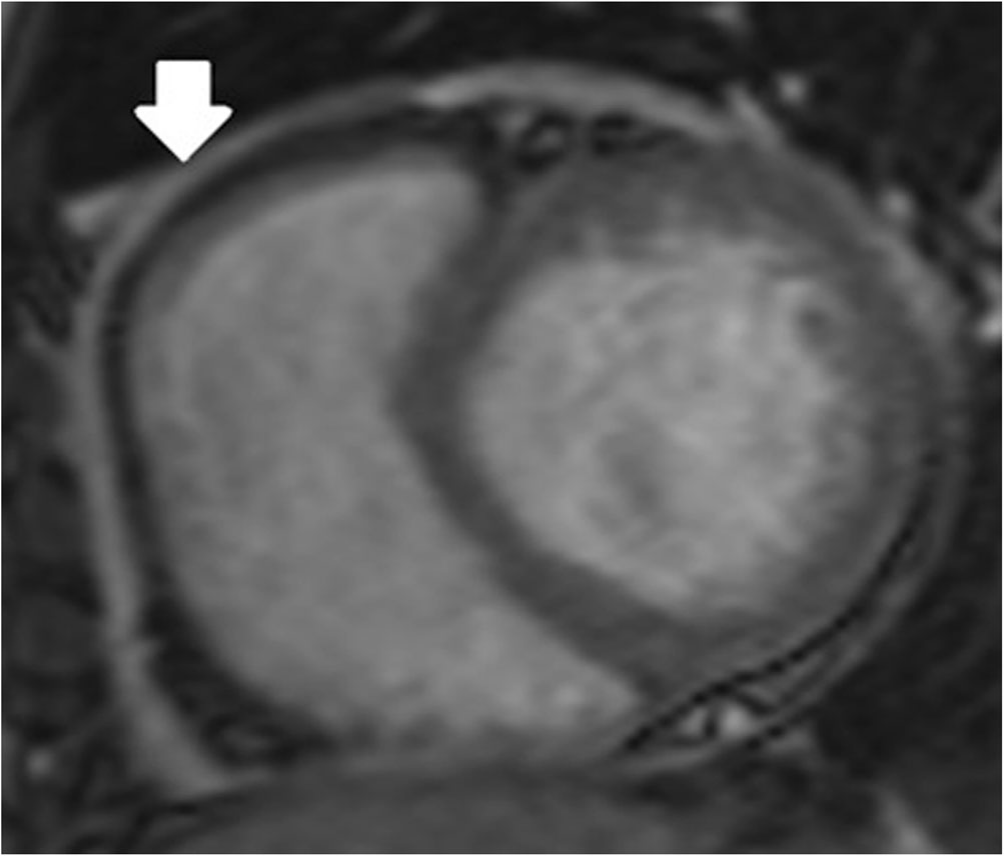
Scout CMR image showing thickened pericardium around the right ventricular free wall (white arrow).

Pericarditis is suspected clinically in this setting when the patient complains of chest pain which is worse on inspiration and better when sitting up. The ECG demonstrates widespread saddle-shaped (concave) ST segment elevation in a non-coronary distribution. There may also be PR segment depression. The patient is often tachycardic and pyrexial. Pericardial inflammation and thickening may be best visualised on cardiac MRI (CMR) (Figs. 1 and 2 respectively). Troponin levels should normal; if elevated, there is myocardial involvement and the diagnosis is one of myopericarditis (see below).

The treatment of pericarditis is usually as per recommended guidelines [13]. However, it is to be recognised that many cancer patients may have a predisposition to bleeding due to abnormal blood counts or coagulation abnormalities secondary to their disease or treatment. It can thus be challenging to introduce routine therapy such as non-steroidal anti-inflammatory agents in this context. As a result, there is often a greater and earlier use of other agents, e.g. colchicine and steroids although this may not alter outcomes [14].

Pericarditis can be complicated by pericardial effusions and tamponade and in the long term by constriction (discussed below).

## Myopericarditis

Myopericarditis is a condition where there is inflammation involving both the pericardium and the myocardium. Myopericarditis can occur acutely after anthracycline administration [15]. High-dose cyclophosphamide can cause acute cardiotoxicity manifest as haemorrhagic myopericarditis [16]. Death has also been reported in this context associated with pericardial effusions and tamponade [17]. Pre-existing cardiac dysfunction, older age, use of other chemotherapeutic agents and type of cancer (e.g. lymphoma) are all risk factors [18]. Renal impairment can increase the risk of cyclophosphamide-related acute myopericarditis [19]. Acute myopericarditis has also been reported in the setting of ATRA (all trans retinoic acid) use for the treatment of acute promyelocytic leukaemia [20, 21•, 22].

Diagnosis is through a combination of clinical examination, laboratory tests and cardiac imaging investigations. The troponin levels are elevated reflecting myocardial damage. There is a role for myocardial and/or pericardial biopsy if the aetiology remains unclear and there is clinical deterioration. However, cardiac biopsies are invasive procedures with a significant degree of procedural risk.

The treatment of myopericarditis in this context depends upon the aetiology, i.e. whether the disease is the cause or the chemotherapy. If it is the former, aggressive treatment of the disease may result in a resolution of cardiac complications. If it is the latter, different chemotherapeutic regimes may need to be used (which may be less effective cancer treatments) with the addition of steroids. In clinical practice, the actual aetiology is often determined through a process of elimination.

## Pericardial effusions and tamponade

Pericardial effusions complicating cancer therapy are not uncommon with 5–15% of cancer patients having a malignant pericardial effusion and 7% having a non-malignant pericardial effusion in a variety of series [23–25]. Any cancer can metastasize to the pericardium resulting in an effusion with the commonest cancers doing so being breast, lung and Hodgkin lymphoma. Mesothelioma is the commonest primary malignant neoplasm while other primary cancers include sarcomas and lymphomas [26•].

**Fig. 3:**
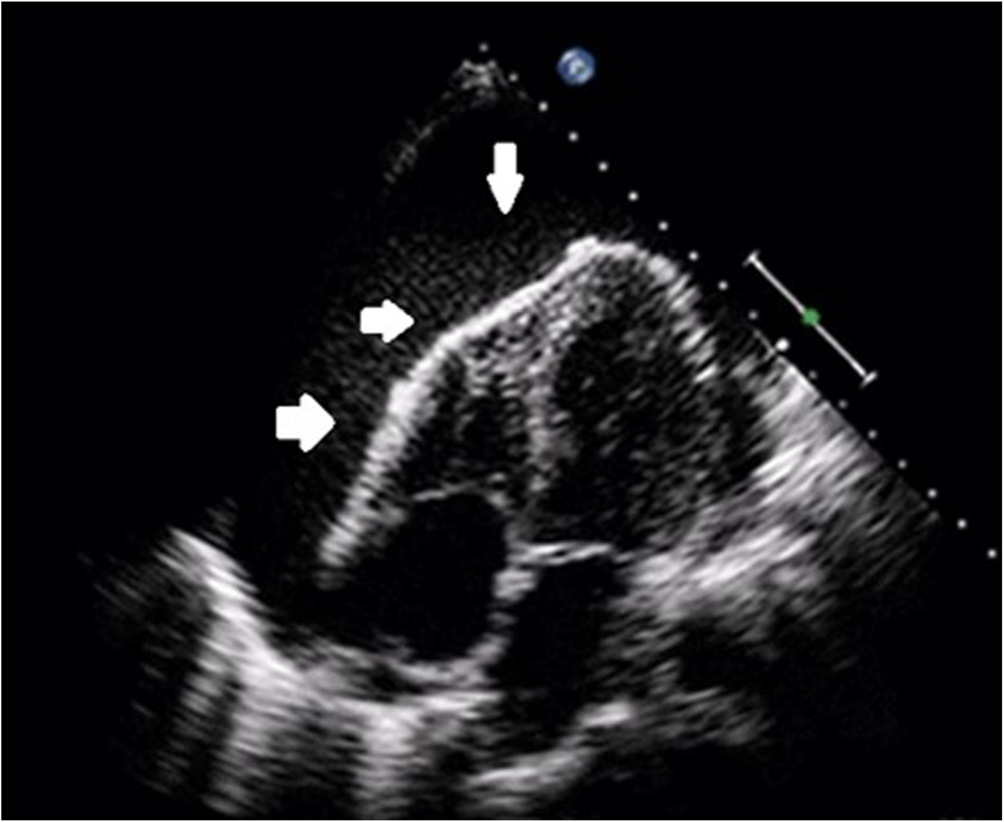
Four-chamber echocardiography still demonstrating large global pericardial effusion (white arrows).

**Fig. 4:**
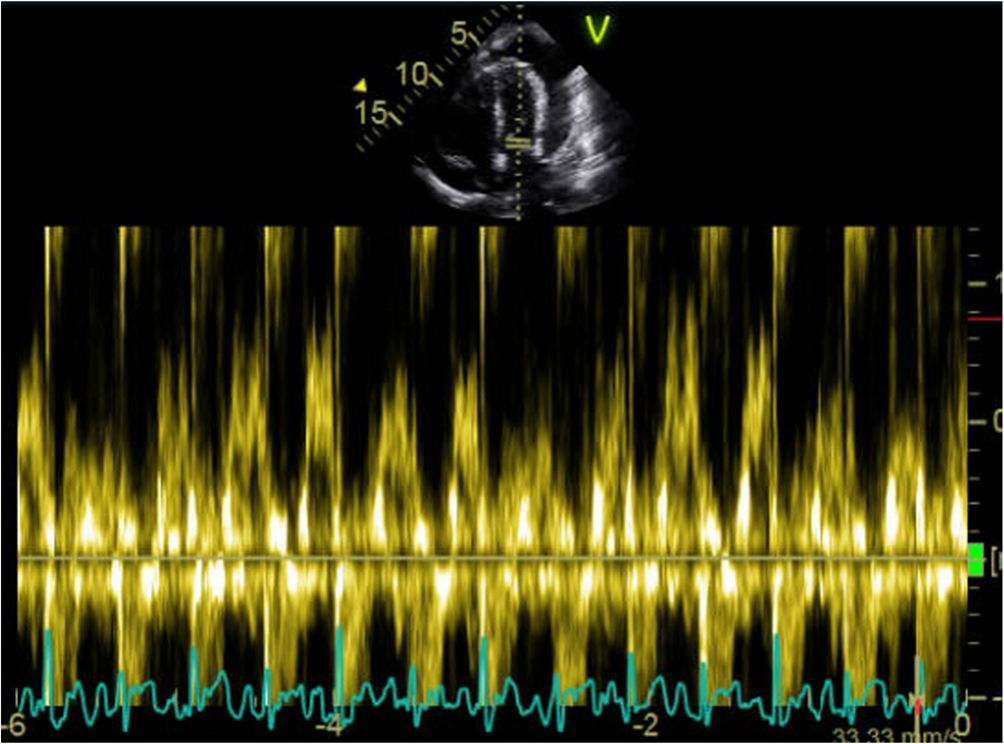
Transmitral Doppler profile demonstrating a > 25% variation in flow with respiration (indicative of tamponade physiology).

Pericardial effusions may or may not be associated with pericarditis and may or may not develop acutely. If they are chronic, they may more often present with a gradually decreasing exercise capacity and an increase in exertional dyspnoea. If the pericardial fluid rapidly accumulates, it may cause cardiac tamponade with acute haemodynamic compromise which requires urgent intervention. Confirmation of the diagnosis is primarily made through echocardiography (Fig. 3) which allows full assessment of the haemodynamic effects of the effusion (Fig. 4) and also allows serial monitoring of the effusion before/after treatment.

The treatment of the effusion depends on the acuteness of symptoms as well as the aetiology. In cases of cardiac tamponade, urgent pericardial drainage is required. In more chronic cases, a decision can be made based on whether the effusion is related to treatment or is malignant. A trial of more intensive chemotherapy or steroids may be undertaken first to determine if this aids effusion resolution. If not, the treatment varies between pericardiocentesis, prolonged pericardial drainage and surgical approaches. It is important to fully evaluate the pericardial fluid no matter which approach is taken. Fluid should be sent for cytology and flow cytometry and consideration should be made to concomitant pericardial biopsy (safer to perform under direct vision in the surgical setting).

Studies have suggested that the surgical treatment of malignant pericardial effusions may offer a more definitive solution to pericardiocentesis [27, 28]. However, such approaches are associated with significant potential morbidity [29•, 30] and recent work has shown that percutaneous approaches may be associated with less morbidity and a low risk of recurrence [31•]. However, a systematic review showed that extended pericardial drainage, pericardial sclerosis and balloon pericardiotomy (10–12% recurrence rate) were all associated with less recurrence than plain pericardiocentesis (38% recurrence rate) [32].

Pericardial sclerosis has been carried out with a variety of agents (tetracycline, bleomycin, talc etc.) but is a painful process. Additionally, a randomised trial showed no statistical difference between this and pericardial drainage in terms of recurrence [33]. Pericardial sclerosis can also lead to pericardial constriction which is a difficult condition to treat (discussed below).

## Pericardial constriction

Pericardial constriction is a condition where there is a loss of the normal elasticity of the pericardial sac. In the context of cancer, this can occur as a result of radiation-induced fibrosis or fibrotic change secondary to pericarditis. A variant is effusive-constrictive pericarditis when pericardial constriction is present along with a pericardial effusion with symptoms and signs of the former often being masked until pericardial drainage is performed.

Clinical signs which while not pathognomonic can aid in diagnosis include pulsus paradoxus (an exaggerated drop in systolic blood pressure of more than 10 mmHg on inspiration), Kussmaul’s sign (lack of an inspiratory decline in JVP) and a pericardial knock (auscultated before an S3). Doppler echocardiography and real-time CMR cines can help in diagnosis. If there remains diagnostic uncertainty, equalisation of left and right ventricular diastolic pressure tracings obtained via cardiac catheterisation reflects increased ventricular interdependence and can clinch the diagnosis.

Symptoms are normally progressive and pericardiectomy remains the most effective therapy. Surgical removal of the pericardium is technically challenging and long-term outcomes are mixed [34]. Outcomes are worse if the pericardial disease was due to radiation therapy [35].

## Tumours of the pericardium

Pericardial tumours include primary and secondary cancers as well as benign lesions (Table 1).

**Table 1:** Commonest primary pericardial tumours (in descending order of prevalence)

Malignant	Benign
Mesothelioma	Cyst
Sarcoma	Lipoma
Lymphoma	Lipoblastoma
	Germ cell tumours
	Paraganglioma
	Fibroma
	Haemangioma

Primary pericardial tumours are rare accounting for around 10% of all primary cardiac tumours [36•, 37] with their prevalence in the general population being 0.001 to 0.007% [26•]. Secondary tumours or direct invasion into the pericardium is around 1000 times more common.

Diagnostically, they can prove a challenge and the definitive diagnosis is often obtained only after pathological analysis of tissue samples. Imaging can help narrow the list of differentials. While CMR is the most adept at tissue characterisation, CT can image tumour invasion of adjacent structures (Table 2). Imaging can also guide the surgeon as to the extent of the operation required and as to whether operative removal is feasible in the place (e.g. in cases where there is tumour encasement of vital structures). Positron emission tomography (PET) scanning can be very helpful in determining whether a particular lesion is active and whether treatment has induced remission.

**Table 2:** Relative strengths and weaknesses of different imaging modalities for pericardial tumours

Imaging modality	Strengths	Weaknesses
Echocardiography	Widely available and portable Relatively inexpensive compared to other techniques Detailed functional and non-invasive haemodynamic assessment Intravenous contrast administration can help determine mass vascularity	Limited tissue characterisation capability Limited information regarding effect on non-cardiac structures
Cardiac magnetic resonance	Non-invasive tissue characterisation Assessment of functional consequence	Unavailability in all centres
Computed tomography	Anatomical delineation of tumour extent Demonstrates effect on adjacent non-cardiac structures	Radiation exposure Limited functional data obtainable
Positron emission tomography	Determines if lesion is metabolically active and guide to whether treatment is effective or not	Radiation exposure Unavailability in all centres

Pericardial cysts (mesothelial cysts) and lipomas are the commonest benign pericardial masses. Pericardial cysts are commonly located at the right (and left) anterior cardiophrenic angles and are commonly asymptomatic. Removal may be required if there are compressive symptoms. Pericardial lipomas are similarly asymptomatic in the majority of cases. Again, symptoms if they occur are related to compression and may in that situation require surgical removal. Other rare and usually benign pericardial tumours include lipoblastomas, paragangliomas, germ cell tumours, haemangiomas and fibromas.

The commonest primary cancer affecting the pericardium is mesothelioma being present in 0.0022% in one autopsy series [38, 39]. It is more common in men than in women and is usually manifested in middle to late age groups. There is no definitive relation to asbestos exposure unlike in pleural mesothelioma. This may manifest as tamponade and constriction [40, 41]. Mesotheliomas can be imaged on echocardiography, cardiac CT and CMR. Resection is the treatment of choice, but the prognosis is poor in metastatic disease [38]. Other pericardial malignancies include lymphomas and sarcomas. Sarcomas are uncommon and subtypes include angiosarcoma, fibrosarcoma, liposarcoma, rhabdomyosarcoma, synovial sarcoma and undifferentiated sarcoma. Prognosis is poor at less than 1 year for all patients. Pericardial lymphomas are usually of the large B cell variety and can often be associated with a pericardial effusion. Metabolic activity is commonly noted on PET imaging.

Pericardial metastasis should be considered when a patient with cancer develops a pericardial effusion or pericarditis. Rarely, pericardial metastases may be the first sign of cancer [42] or may be found incidentally in asymptomatic individuals [26•]. The investigations performed are similar to those for primary pericardial neoplasms.

## Summary

Cancer can affect the pericardium in a variety of ways either as a result of primary or secondary disease or as a result of chemotherapy or radiotherapy. The aetiology of pericardial disease in this setting can often be difficult to determine which may result in a delay in optimal treatment being instituted. Despite optimal treatment, some conditions may recur, e.g. pericardial effusions or have a poor prognosis, e.g. constrictive pericarditis and mesothelioma.
